# Using functional connectivity models to characterize relationships between working and episodic memory

**DOI:** 10.1002/brb3.2105

**Published:** 2021-06-17

**Authors:** Gigi F. Stark, Emily W. Avery, Monica D. Rosenberg, Abigail S. Greene, Siyuan Gao, Dustin Scheinost, R. Todd Constable, Marvin M. Chun, Kwangsun Yoo

**Affiliations:** ^1^ Department of Psychology Yale University New Haven CT USA; ^2^ Department of Psychology University of Chicago Chicago IL USA; ^3^ Interdepartmental Neuroscience Program Yale University New Haven CT USA; ^4^ Department of Biomedical Engineering Yale University New Haven CT USA; ^5^ Department of Diagnostic Radiology Yale School of Medicine New Haven CT USA; ^6^ Department of Neurosurgery Yale School of Medicine New Haven CT USA; ^7^ Department of Neuroscience Yale School of Medicine New Haven CT USA

**Keywords:** episodic memory, functional connectivity, *N*‐back, predictive model, working memory

## Abstract

**Introduction:**

Working memory is a critical cognitive ability that affects our daily functioning and relates to many cognitive processes and clinical conditions. Episodic memory is vital because it enables individuals to form and maintain their self‐identities. Our study analyzes the extent to which whole‐brain functional connectivity observed during completion of an *N*‐back memory task, a common measure of working memory, can predict both working memory and episodic memory.

**Methods:**

We used connectome‐based predictive models (CPMs) to predict 502 Human Connectome Project (HCP) participants' in‐scanner 2‐back memory test scores and out‐of‐scanner working memory test (List Sorting) and episodic memory test (Picture Sequence and Penn Word) scores based on functional magnetic resonance imaging (fMRI) data collected both during rest and *N*‐back task performance. We also analyzed the functional brain connections that contributed to prediction for each of these models.

**Results:**

Functional connectivity observed during *N*‐back task performance predicted out‐of‐scanner List Sorting scores and to a lesser extent out‐of‐scanner Picture Sequence scores, but did not predict out‐of‐scanner Penn Word scores. Additionally, the functional connections predicting 2‐back scores overlapped to a greater degree with those predicting List Sorting scores than with those predicting Picture Sequence or Penn Word scores. Functional connections with the insula, including connections between insular and parietal regions, predicted scores across the 2‐back, List Sorting, and Picture Sequence tasks.

**Conclusions:**

Our findings validate functional connectivity observed during the *N*‐back task as a measure of working memory, which generalizes to predict episodic memory to a lesser extent. By building on our understanding of the predictive power of *N*‐back task functional connectivity, this work enhances our knowledge of relationships between working memory and episodic memory.

## Graphical abstract

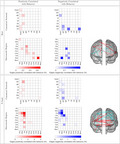

## 1 INTRODUCTION

Working memory is the ability to retain a limited quantity of information and put it to use in cognitive tasks (Cowan, 2014). Our daily functioning relies heavily on working memory, and working memory capacity, a construct used to connote differences in individuals' working memory capabilities (Wilhelm et al., 2013). Working memory capacity is related to other cognitive abilities including problem solving (Wiley & Jarosz, 2012), reading comprehension (Daneman & Carpenter, 1980), reasoning (Kyllonen & Christal, 1990), controlled attention (Engle et al., 1999), and fluid intelligence (Colom et al., 2015). Furthermore, working memory capacity is impaired in a number of psychiatric and neurodevelopmental conditions, including schizophrenia (Gold et al., 2003, 2006), attention deficit disorder (Alderson et al., 2013), and reading disabilities (Gathercole et al., 2006). Episodic memory is a form of declarative memory that focuses on the ability to recall events tied to a specific place and time (Dikmen et al., 2014). This type of memory is critical because it helps individuals build and maintain their self‐identities (Dikmen et al., 2014). To further our understanding of relationships between working and episodic memory, our study seeks to determine the extent to which functional connectivity observed during performance of an *N*‐back working memory task reflects individual differences in both working memory and episodic memory.

In neuroimaging research, the *N*‐back task is a common test of working memory (Jaeggi et al., 2010). The *N‐*back test, first introduced by Kirchner (1958), targets memory by requiring participants to recognize the item presented *n* items back. This test is popular because varying *n* is an easy way to manipulate working memory loads (Jaeggi et al., 2010) and because the test's administration and response requirements are not overly complex (Conway et al., 2003). Although the *N*‐back task measures aspects of working memory, work has suggested that performance on this task does not reflect working memory capacity alone. For example, Kane et al. (2007) and Jaeggi et al. (2010) both found that performance on the *N*‐back task is related to both working memory abilities and fluid intelligence.

Prior studies have found a relationship between working and episodic memory. Lugtmeijer et al. (2019) found a significant correlation (*r* = .504, *p* = .005) between the 2‐back working memory test scores and the subsequent episodic memory test scores of 29 adults ages 20–29. This finding suggests a relationship between the *N*‐back test and episodic memory for young adults. Other research also has established relationships between individual differences in episodic memory and working memory. Hertzog et al. (2003) found that for 303 adults ages 61–91, over 6 years, changes in episodic memory were significantly correlated with changes in working memory and that these shifts could best be explained by changes in induction and fact retrieval. In two different experiments that each examined distinct sets of twenty individuals (between ages 18 and 35) with poor working memory capabilities, Unsworth (2007) found that those with lower working memory capacity also experienced deficits in episodic retrieval and that these individuals struggle with episodic retrieval in part because they search through more items than those with strong working memory capacity do. Unsworth et al. (2011), which explored how encoding specificity affects the relationship between an individual's episodic recall and working memory capacity, deduced that the conditions surrounding an episodic recall task affect the correlation between performance on the task and working memory capacity. For a sample of 11,537 9–10‐year‐olds, Rosenberg, Martinez, et al. (2020) found a sizable Spearman correlation between List Sorting and Picture Sequence memory test scores (*r* = .34) and between 2‐back and Picture Sequence memory test scores (*r* = .31). We use 2‐back and List Sorting scores to operationalize working memory and Picture Sequence scores to represent episodic memory.

Prior work has established that functional connectivity, measured by functional magnetic resonance imaging (fMRI) scans, can be an effective metric to predict individual differences in cognitive abilities and behavior. Finn et al. (2015) found that every individual has a unique pattern of functional connectivity that can be measured either at rest or during a cognitive task. Previous work has discovered relationships between functional connectivity and numerous cognitive abilities, such as attention (Rosenberg et al., 2016; Yoo et al., 2018), impulsivity (Li et al., 2013), and intelligence (Finn et al., 2015; Hearne et al., 2016; van den Heuvel et al., 2009; Yoo et al., 2019). Critically, functional connectivity can be used to predict memory capabilities, as previous work has established relationships between functional connectivity and both working memory (Avery et al., 2020) and Alzheimer's‐related cognitive impairment (Lin et al., 2018). Avery et al. (2020) found that functional connectivity observed during both rest and *N*‐back task performance predicted in‐scanner 2‐back task performance.

Building on Avery et al. (2020), we evaluate the extent to which *N*‐back task functional connectivity reflects different types of memory by measuring how well fMRI data collected during an *N*‐back task predicts several out‐of‐scanner memory test scores. To evaluate whether these trends are specific to *N*‐back task functional connectivity or generalize to rest functional connectivity, we also measure how well fMRI data collected at rest predicts the same memory test scores. Using Human Connectome Project (HCP) fMRI data and memory test scores, we analyze the extent to which *N*‐back task functional connectivity captures individual differences in both working memory and episodic memory by evaluating: (a) the extent to which resting‐state and *N*‐back task functional connectivity can predict out‐of‐scanner List Sorting, Picture Sequence, and Penn Word scores, and (b) the commonalities between the features predicting in‐scanner 2‐back task performance and those predicting out‐of‐scanner List Sorting, Picture Sequence, and Penn Word memory test performance. Both the *N*‐back and List Sorting (Tulsky et al., 2014) tests are considered measures of working memory, whereas the Picture Sequence (Dikmen et al., 2014) and Penn Word (Gur et al., 2001, 2010) tests capture episodic memory. Thus, we hypothesize that *N*‐back task functional connectivity will better predict List Sorting than Picture Sequence or Penn Word memory test scores, and that the functional connections that predict 2‐back task performance will be more similar to those that predict List Sorting scores than to those that predict Picture Sequence or Penn Word scores.

## 2 METHODS

### 2.1 Data

We obtained rest and *N*‐back task fMRI data and behavioral task performance data from the December 2015 HCP 900‐participant release (Van Essen et al., 2013). These scans had a 3T scanner magnetic field, a multiband factor (slice acceleration factor) of 8, no in‐plane phase encode acceleration, a spatial resolution of 2 mm isotropic, a 33 ms delay between signal excitation and image acquisition time (echo time), and a .72 s volume repetition time (Uğurbil et al., 2013). We used the same 502‐participant subset of this data set as Avery et al. (2020) and thus build on this work. Each of these participants (mean age 28 ± 3.6 years; 274 females) completed all nine HCP fMRI conditions and across these conditions had a grand mean frame‐to‐frame head motion less than .10 mm and a maximum frame‐to‐frame motion less than .15 mm (Greene et al., 2018). Head motion from left/right and right/left phase‐encoding runs was averaged to obtain average frame‐to‐frame motion for each fMRI task. Each of these participants also had 2‐back, List Sorting, Picture Sequence, and Penn Word scores available and was not missing any functional atlas nodes or time points. All participants provided written consent per the regulations of the following institutions: Washington University in Saint Louis, University of Minnesota, Oxford University, Saint Louis University, Indiana University, University d'Annunzio, Ernst Strüngmann Institute, Warwick University, Radboud University Nijmegen, and Duke University.

BioImage Suite (Joshi et al., 2011) and minimally preprocessed HCP data (Glasser et al., 2013) were used to create functional connectivity matrices that served as input to our connectome‐based predictive models (CPMs) (Finn et al., 2015; Rosenberg et al., 2016; Shen et al., 2017). These matrices represent the magnitude of functional connectivity between predefined nodes encompassing the entire brain. In generating these connectivity matrices, we first regressed a series of nuisance covariates out of the fMRI data, removed the linear trend, and performed low‐pass filtering. These nuisance covariates included 12 motion parameters and white matter, cerebral spinal fluid, and global signals. For low‐pass filtering, data were temporally smoothed with a Gaussian filter (mean = 0, variance = 2.17, cutoff frequency = .12 Hz). We divided the brain into 268 regions defined in volumetric space (Shen et al., 2013) and extracted the mean fMRI signal time series at each of these nodes. We defined regions in volumetric space because we wanted to include subcortical regions and replicate previous CPM work, particularly Avery et al. (2020) on which this study builds, that used a volumetric Shen 268‐node atlas. We obtained connectivity matrix entries by calculating the Pearson correlation coefficients between each pair of time series and then converting these *r*‐values to *z*‐values using the Fisher transform.

We used fMRI data collected both while participants were at rest and while they performed two five‐minute *N*‐back memory task runs. The *N*‐back task required participants to complete 2‐back and 0‐back memory tests, each 50% of the time. Participants were presented with pictures of four different types of stimuli: places, tools, faces, and body parts. Each run consisted of eight task blocks and four resting fixation blocks. Each task block consisted of a specific memory test (0‐back or 2‐back) for 10 images of one stimulus type. Additional details regarding this task can be found in Barch et al. (2013).

We examined individual variation in performance on Form A of the Penn Word memory test (Gur et al., 2001, 2010). This test was designed to measure verbal episodic memory. Participants were first asked to remember 20 words. After 20 minutes, participants were given 40 words, which included the 20 original words and 20 new words. Participants were asked to guess whether each word was new or part of the original set. We analyzed each participant's number of correct responses for this task.

For the Picture Sequence memory test, we quantified participants' abilities to remember increasingly long series of pictures and the order in which the pictures were presented. In this National Institutes of Health (NIH) Toolbox test (Dikmen et al., 2014), participants were shown a number of pictures and given one point for each correctly ordered pair of pictures. Most episodic memory tests largely depend upon verbal skills, whereas the Picture Sequence test was specifically designed to measure episodic memory in young children who lack verbal skills.

For the List Sorting memory test (Tulsky et al., 2014), we quantified participants' abilities to recall and manipulate sequences of pictures. Also an NIH Toolbox assessment, this test presented participants with increasingly long series of pictures of food and animals. As part of this test, participants completed two tasks. In the first task, participants were shown pictures of either food or animals and then were asked to recall the objects and order them from smallest to largest. In the second task, participants were shown pictures of both food and animals and then were asked to recall the objects and separately order the animal pictures and the food pictures, each based on the size of the depicted objects. Each participant's final score was determined based on the number of correct sorts across both tasks. The List Sorting memory test is a sequencing task, a task‐type which has proven successful in measuring working memory in many previous works (Gold et al., 1997; Mungas et al., 2000; Tulsky et al., 2003; Wechsler, 1997).

### 2.2 Measuring the similarity between memory tests

We calculated correlations between participants' performance on different memory tests to verify whether the CPM and functional anatomy analyses reflect inherent similarities between memory tests. We calculated the correlations between each pair of observed 2‐back, Penn Word, Picture Sequence, and List Sorting scores. For each of these correlations, we reported a parametric *p*‐value. Using Steiger's *z*‐tests to compare pairs of correlations, we evaluated whether 2‐back task performance was more closely related to List Sorting test performance than to Picture Sequence or Penn Word test performance. To correct for multiple comparisons, for each *p*‐value in both our Steiger's *z*‐test and CPM analyses, we applied a Bonferroni correction to the original significance threshold (*p* < .05).

### 2.3 Connectome‐based predictive modeling

Using the R programming language (version 3.4.4), for both rest and *N*‐back task functional connectivity, we constructed four CPMs to predict the 2‐back, List Sorting, Picture Sequence, and Penn Word memory test scores of the 502 examined HCP participants. We performed this analysis with rest functional connectivity to determine whether the patterns of results observed for *N*‐back task functional connectivity analysis generalized to functional connectivity observed in the absence of an explicit task. We predicted participants' performance on only the 2‐back memory test portion of the *N*‐back task because the 2‐back test (mean accuracy = 84.7%, standard deviation of accuracy = 10.4%) taxes working memory more heavily than does the 0‐back test (mean accuracy = 91.4%, standard deviation of accuracy = 10.1%). The 2‐back and List Sorting tasks are considered working memory measures, whereas the Penn Word and Picture Sequence tasks target episodic memory. Thus, predicting these memory test scores based on fMRI data collected during an *N*‐back task allowed us to characterize the extent to which functional connectivity measured during an *N*‐back task captures both working and episodic memory.

CPM, a type of recently developed brain‐based prediction approach, identifies key connections from whole‐brain functional connectivity and uses these connections to predict variation in a behavior or trait across participants (Finn et al., 2015; Rosenberg et al., 2016; Shen et al., 2017). For our study, the predicted behavior was performance on a memory test (2‐back, Penn Word, Picture Sequence, or List Sorting). For each type of functional connectivity (rest and *N*‐back task), we constructed separate CPMs for each of the four memory tests examined.

The first step in training our CPMs was edge selection, or identification of the specific functional connections that were most related to behavior in the training sample. We selected connections that were either positively or negatively correlated (at a .01 *p*‐value threshold) with the measured behavior. In selecting connectivity features, we used a partial correlation to control for participants' average frame‐to‐frame head motion. In other words, during feature selection, we correlated the strength of every connection with behavior in the training sample, controlling for frame‐to‐frame head motion.

Our edge selection step allowed us to create what we define as a mask, the set of edges, or connections between brain nodes, that are significantly correlated with the examined behavior. Edges significantly positively correlated with behavior constituted what we call the positive mask, and edges significantly negatively correlated with behavior comprised the negative mask. For each training data set participant, we then computed the mean positive mask edge weight and the mean negative mask edge weight by averaging the strengths of the positive mask edges and negative mask edges for that particular participant. Using the R MASS package's “rlm” function (Venables & Ripley, 2002), we built a robust linear regression with a bisquare weighting function to relate memory test scores to the difference between average positive mask and average negative mask edge weights in the training set. For model validation, we used the training data set's positive and negative masks to calculate this input for testing data set participants. We then input these values into the trained robust linear regression to predict the behavior of each testing data set participant. Of note, positive and negative networks were defined separately to allow us to interpret the anatomy of networks positively and negatively related to behavioral scores. Including collinear predictors in a regression model can cause model parameter estimates to be unreliable (Alin, 2010). To avoid collinear predictors, a single value (mean connectivity strength in the positive network minus mean connectivity strength in the negative network) was input into the linear model to generate behavioral predictions, as described in previous work (Avery et al., 2020; Rosenberg, Scheinost, et al., 2020).

We measured the ability of the *N*‐back task functional connectivity to predict each score by calculating the correlations between CPM‐predicted and observed Penn Word, Picture Sequence, and List Sorting scores, and the correlations between CPM‐predicted 2‐back scores and observed Penn Word, Picture Sequence, and List Sorting scores. We similarly evaluated the extent to which fMRI scan data collected at rest predicted List Sorting, Picture Sequence, and Penn Word scores.

We trained and tested our CPMs using 10‐fold cross‐validation. For each combination of functional connectivity (rest and *N*‐back task) and memory test score (2‐back, List Sorting, Picture Sequence, and Penn Word), we ran 1,000 iterations of 10‐fold cross‐validation in order to obtain reliable estimates of CPM performance. For each iteration, we divided participants into 10 roughly equal folds, ensuring that members of the same family were in the same fold. In generating these folds, we first randomly assigned each family to a fold. If any fold had more than 60 participants, we moved all members of the largest fold's largest family to the smallest fold. We repeated this process until we had no folds with more than 60 participants. We predicted the memory test scores of each fold's participants using a CPM trained on the other nine folds of data. For a particular iteration, the same 10‐fold cross‐validation split was used for CPMs predicting 2‐back, Penn Word, Picture Sequence, and List Sorting scores. In evaluating CPM performance, we reported average correlations between CPM‐predicted and observed scores across 1,000 iterations of 10‐fold cross‐validation. To calculate a non‐parametric *p*‐value for every average correlation, we ran 1,000 iterations of 10‐fold cross‐validation null CPM models for each functional connectivity/memory test score pair. We defined a null CPM model as a CPM for which the order of participant connectivity matrices remained the same, but the memory test scores were randomly shuffled. For a particular iteration, the same 10‐fold cross‐validation split was used for null CPMs predicting 2‐back, Penn Word, Picture Sequence, and List Sorting memory test scores. We calculated null correlations, which were correlations between null CPM‐predicted and observed memory test scores. For each actual model average correlation, the *p*‐value was calculated as *p* = (1 + the number of null correlations greater than or equal to the actual model average correlation)/(1 + the total number of iterations).

For both rest and *N*‐back task functional connectivity, we tested whether predictive power for List Sorting scores was statistically different from predictive power for Picture Sequence or Penn Word scores. For each of the two metrics of CPM performance (correlations between predicted and observed Penn Word, Picture Sequence, and List Sorting scores, and correlations between predicted 2‐back and observed Penn Word, Picture Sequence, and List Sorting scores), we separately evaluated these differences. For each combination of functional connectivity type and CPM performance metric, we separately examined the distribution of the differences between each iteration's List Sorting and Picture Sequence correlations and the distribution of the differences between each iteration's List Sorting and Penn Word correlations. Each of these distributions contained 1,000 correlation differences from 1,000 iterations. For each distribution of correlation differences, we performed a t‐test to determine whether this distribution was significantly different from the distribution of the differences between the null model correlations for the same memory test scores. For example, for CPMs with *N*‐back task functional connectivity as input, we compared: (a) the distribution of the differences of the correlations between CPM‐predicted and observed List Sorting scores minus the correlations between CPM‐predicted and observed Picture Sequence scores to (b) the distribution of the differences of the correlations between null CPM‐predicted and observed List Sorting scores minus the correlations between null CPM‐predicted and observed Picture Sequence scores. Through these analyses, for both rest and *N*‐back task functional connectivity, we determined whether the difference in the ability of the connectivity matrices to predict one memory test versus another was statistically significant.

### 2.4 Analyzing the functional anatomy of predictive networks

To characterize the connections used by 2‐back, Penn Word, Picture Sequence, and List Sorting models, we analyzed the edges between each pair of 268 brain nodes (Shen et al., 2013) used to predict each memory test score. For each combination of functional connectivity (rest and *N*‐back task) and memory test score (2‐back, List Sorting, Picture Sequence, and Penn Word), we trained a CPM on data from all 502 participants and tracked the identified predictive positive mask and predictive negative mask connectivity features.

To measure the similarity between the predictive positive mask connectivity features of the 2‐back and those of each other memory test score, we calculated the percentage of overlapping edges between the positive mask used to predict the 2‐back score and the positive mask used to predict each other score. Similarly, we calculated the percentage of overlapping edges between the predictive negative masks of the 2‐back and each other memory test score.

Additionally, based on results from the CPM trained on data from all 502 participants, we visualized the functional connections that predicted each memory test score. We grouped the 268 brain nodes (Shen et al., 2013) into 8 functional canonical networks and 10 anatomic macroscale brain regions. Each connection between canonical networks and each connection between macroscale regions consisted of many edges between these 268 brain nodes. Canonical networks were defined as in Finn et al. (2015) and included the default mode, subcortical cerebellum, frontoparietal, motor, medial frontal, visual association, VI, and VII. The selected macroscale regions were a variety of cortical and subcortical brain regions, including the prefrontal cortex, motor cortex, insula, parietal, temporal, occipital, limbic, cerebellum, subcortical, and brainstem.

For each combination of functional connectivity (rest and *N*‐back task) and memory test score (2‐back, Penn Word, Picture Sequence, and List Sorting), we visualized the contribution of each canonical network and macroscale region to predictive networks. Additionally, for both rest and *N*‐back task functional connectivity, we visualized the connections that consistently predicted 2‐back, List Sorting, and Picture Sequence scores—that is, the connections that predicted all three of these measures of memory.

### 2.5 Controlling for age in connectome‐based predictive modeling

To test whether controlling for age, a potential confounding factor (Small, 2001), altered results, we performed analyses with the HCP's age‐adjusted versions of two types of memory test scores. The HCP provided age‐adjusted versions of both the Picture Sequence and List Sorting scores, but not the Penn Word or 2‐back scores. The memory test scores for which the HCP offered age‐adjusted versions were NIH Toolbox scores, which were age‐adjusted using NIH Toolbox normative data (Barch et al., 2013). As the Penn Word and 2‐back scores are not NIH Toolbox scores, equivalent normative data were not available, preventing us from generating age‐adjusted versions of these scores. We calculated the correlations between each pair of observed 2‐back, Penn Word, age‐adjusted List Sorting, and age‐adjusted Picture Sequence scores. We completed both CPM and functional anatomy analyses for the age‐adjusted Picture Sequence and age‐adjusted List Sorting scores. These analyses were comparable to those completed for the unadjusted versions of these scores.

## 3 RESULTS

### 3.1 Measuring the similarity between memory tests

#### 3.1.1 Comparing correlations between 2‐back and other observed memory test scores

For the studied HCP participants, we determined that the correlation between the observed 2‐back and List Sorting scores was greater than both the correlation between the observed 2‐back and Penn Word scores and the correlation between the observed 2‐back and Picture Sequence scores (see Table 1). At a *p* < .05 significance level corrected for multiple comparisons, Steiger's *z*‐tests (using two‐sided *p*‐values) found that these differences were statistically significant for both the List Sorting/Penn Word comparison (*z* = 3.2 and *p* = 1.3e−3) and the List Sorting/Picture Sequence comparison (*z* = 2.4 and *p* = 1.7e−2). When we examined age‐adjusted Picture Sequence and age‐adjusted List Sorting scores, for the same significance threshold, the correlation between the 2‐back and List Sorting scores was significantly greater than the correlation between the 2‐back and Penn Word scores and marginally significantly greater than the correlation between the 2‐back and Picture Sequence scores (see Tables S1 and S2).

**TABLE 1:** Correlations between observed memory test scores

	Penn Word	Picture Sequence	List Sorting
2‐back	.20 (*p* = 5.2e−6)	.27 (*p* = 1.4e−9)	.38 (*p* < 2.2e−16)
Penn Word	—	.20 (*p* = 4.1e−6)	.09 (*p* = 4.5e−2)
Picture Sequence	—	—	.30 (*p* = 6.2e−12)

Note

The correlations between the observed 2‐back, Penn Word, Picture Sequence, and List Sorting memory test scores of the 502 examined Human Connectome Project (HCP) participants.

#### 3.1.2 Evaluating the correlations between observed List Sorting, Picture Sequence, and Penn Word memory test scores

To identify the overlap in represented constructs across the memory tests, we evaluated the correlations between each pair of observed List Sorting, Picture Sequence, and Penn Word memory test scores. We found a relatively large correlation between the observed List Sorting and Picture Sequence scores (*r* = .30, *p = *6.2e−12), and a much smaller correlation between the observed List Sorting and Penn Word scores (*r* = .09, *p* = 4.5e−2) (see Table 1). The correlation between the observed Picture Sequence and Penn Word memory test scores (*r* = .20, *p* = 4.1e−6), the two episodic memory measures, was relatively small (see Table 1). The correlations between the Penn Word, age‐adjusted Picture Sequence, and age‐adjusted List Sorting scores were relatively similar to those between all unadjusted scores (see Table S1).

### 3.2 Analyzing connectome‐based predictive models predicting different memory test scores

#### 3.2.1 Evaluating model performance for different memory test scores

We examined two different metrics of CPM performance: correlations between predicted and observed Penn Word, Picture Sequence, and List Sorting scores, and correlations between predicted 2‐back and observed Penn Word, Picture Sequence, and List Sorting scores.

**FIGURE 1:**
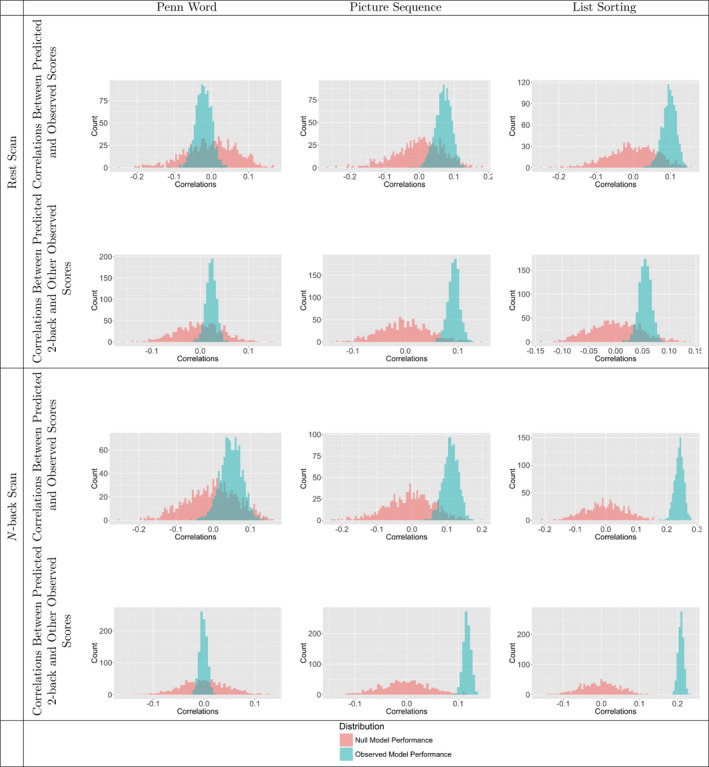
Histograms comparing correlations between predicted and observed memory test scores for actual and null connectome‐based predictive models. For both rest and *N*‐back task functional connectivity and for iterations of both actual and null connectome‐based predictive models (CPMs), each of the 1,000 correlations between predicted and observed Penn Word, Picture Sequence, and List Sorting scores and each of the 1,000 correlations between predicted 2‐back and observed Penn Word, Picture Sequence, and List Sorting scores.

Using *N*‐back task connectivity matrices as input, CPMs significantly predicted List Sorting (working memory) scores, and marginally predicted Picture Sequence (episodic memory) scores. At a *p* < .05 significance level corrected for multiple comparisons, we observed a significant correlation between the CPM‐predicted and observed List Sorting scores and a marginally significant correlation between the CPM‐predicted and observed Picture Sequence scores (see Table 2 and Figure 1). At the same significance level, we saw significant correlations between the CPM‐predicted 2‐back scores and both the observed Picture Sequence scores and the observed List Sorting scores (see Table 3 and Figure 1). Neither the correlation between the CPM‐predicted and observed Penn Word (episodic memory) scores nor the correlation between the CPM‐predicted 2‐back and observed Penn Word scores were significant (see Tables 2 and 3 and Figure 1).

For List Sorting and Picture Sequence memory test score prediction, the results of the resting‐state functional connectivity models did not fully replicate those of the *N*‐back task functional connectivity models, but both rest and *N*‐back task functional connectivity poorly predicted Penn Word scores (see Tables 2 and 3 and Figure 1). Whereas *N*‐back task functional connectivity predicted List Sorting scores and marginally predicted Picture Sequence scores, rest functional connectivity marginally predicted List Sorting scores and did not significantly predict Picture Sequence scores.

**TABLE 2:** Correlations between predicted and observed memory test scores

Training & test behavior	Models trained and tested using resting‐state functional connectivity			Models trained and tested using *N*‐back task functional connectivity		
	Mean *r*‐value	Standard deviation *r‐*value	*p*‐value	Mean *r*‐value	Standard deviation *r‐*value	*p*‐value
2‐back	.20^a^		1/1,001^a^	.36^a^		1/1,001^a^
Penn Word	−.02	.02	.62	.05	.03	.24
Picture Sequence	.07	.02	.15	.11	.02	3.5e−2
List Sorting	.10	.02	4.8e−2	.24	.01	1.0e−3

Note

The correlations between the predicted and observed Penn Word, Picture Sequence, and List Sorting memory test scores of the 502 examined Human Connectome Project (HCP) participants.

^a^ These numbers were taken from Avery et al. (2020), which for the same set of subjects as our work, used the same rest and *N*‐back task functional connectivity and 10‐fold cross‐validation to predict 2‐back memory test scores.

**TABLE 3:** Correlations between predicted 2‐back and other observed memory test scores

Observed behavior	Models trained and tested using resting‐state functional connectivity			Models trained and tested using *N*‐back task functional connectivity		
	Mean *r*‐value	Standard deviation *r‐*value	*p*‐value	Mean *r*‐value	Standard deviation *r‐*value	*p*‐value
Penn Word	.02	.01	.31	−1.6e−3	7.4e−3	.51
Picture Sequence	.09	.01	2.5e−2	.12	.01	3.0e−3
List Sorting	.05	.01	.11	.21	.01	1.0e−3

Note

The correlations between the predicted 2‐back and the observed Penn Word, Picture Sequence, and List Sorting memory test scores of the 502 examined Human Connectome Project (HCP) participants.

The rest and *N*‐back task functional connectivity analyses that used age‐adjusted List Sorting and age‐adjusted Picture Sequence scores demonstrated similar trends to the comparable analyses that used the unadjusted versions of these scores (see Tables S3 and S4 and Figure S1). Overall, these results suggest that *N*‐back task functional connectivity captures not only working memory, but also episodic memory capabilities.

#### 3.2.2 Comparing model performance for different memory test scores

Our *t*‐tests supported the hypothesis that at a *p* < .05 significance level corrected for multiple comparisons, *N*‐back task functional connectivity predicted List Sorting scores significantly better than it predicted Picture Sequence or Penn Word scores (see Tables 4 and 5 and Figure S2). For both metrics of CPM performance and for comparisons of List Sorting correlations both to Penn Word correlations and to Picture Sequence correlations, each *t*‐test produced a *p*‐value that was less than 2.2e−16. For each comparison, the 97.5% confidence interval contrasting the difference distribution and its corresponding null difference distribution did not contain 0.

Rest functional connectivity comparison analyses produced different results from the comparable *N*‐back task analyses (see Tables 4 and 5 and Figure S2). For example, for *N*‐back task functional connectivity, the correlation between predicted 2‐back scores and observed List Sorting scores was significantly stronger than the correlation between CPM‐predicted 2‐back scores and observed Picture Sequence scores. In contrast, for rest functional connectivity, the correlation between predicted 2‐back scores and observed Picture Sequence scores was significantly stronger than the correlation between predicted 2‐back scores and observed List Sorting scores.

**TABLE 4:** Comparing correlations between predicted and observed memory test scores

Training & test behaviors	Models trained and tested using resting‐state functional connectivity			Models trained and tested using *N*‐back task functional connectivity		
	*t*‐value	*p*‐value	97.5% confidence interval	*t*‐value	*p‐*value	97.5% confidence interval
Penn Word/List Sorting	37.5	<2.2e−16	(.11, .13)	58.6	<2.2e−16	(.18, .20)
Picture Sequence/List Sorting	9.6	<2.2e−16	(.02, .04)	42.8	<2.2e−16	(.12, .13)

Note

The *t*‐test results for comparing difference distributions for the correlations between predicted and observed memory test scores to the corresponding null difference distributions. Each actual and each null difference distribution equals a distribution of correlations between predicted and observed List Sorting scores minus correlations between predicted and observed Picture Sequence or Penn Word scores. Thus, a significant positive *t*‐value indicates that List Sorting scores were predicted significantly better than Picture Sequence or Penn Word scores. A significant negative *t*‐value indicates that Picture Sequence or Penn Word scores were predicted significantly better than List Sorting scores.

**TABLE 5:** Comparing correlations between predicted 2‐back and other observed memory test scores

Observed behaviors	Models trained and tested using resting‐state functional connectivity			Models trained and tested using *N*‐back task functional connectivity		
	*t*‐value	*p*‐value	97.5% confidence interval	*t*‐value	*p‐*value	97.5% confidence interval
Penn Word/List Sorting	15.7	<2.2e−16	(.03, .04)	109.1	<2.2e−16	(.21, .22)
Picture Sequence/List Sorting	−21.3	<2.2e−16	(−.04, −.03)	52.9	<2.2e−16	(.09, .10)

Note

The *t*‐test results for comparing difference distributions for the correlations between predicted 2‐back and other observed memory test scores to the corresponding null difference distributions. Each actual and each null difference distribution equals a distribution of correlations between predicted 2‐back and observed List Sorting scores minus correlations between predicted 2‐back and observed Picture Sequence or Penn Word scores. Thus, a significant positive *t*‐value indicates that CPM‐predicted 2‐back scores are significantly more similar to observed List Sorting scores than to observed Picture Sequence or Penn Word scores. A significant negative *t*‐value indicates that CPM‐predicted 2‐back scores are significantly more similar to observed Picture Sequence or Penn Word scores than to observed List Sorting scores.

The rest and *N*‐back task comparison analyses that used age‐adjusted List Sorting and age‐adjusted Picture Sequence scores achieved similar results (see Tables S5 and S6 and Figure S3).

### 3.3 Analyzing the functional anatomy of models predicting different memory test scores

#### 3.3.1 Measuring the percentage of overlapping edges used to predict 2‐back and other memory test scores

For both rest and *N*‐back task connectivity matrices, our analyses of the functional anatomy of our CPMs found that the connections used in 2‐back score prediction were most similar to those used in List Sorting score prediction, second most similar to those used in Picture Sequence score prediction, and least similar to those used in Penn Word score prediction. For both rest and *N‐*back task functional connectivity and for both positive and negative masks, the percentage of overlapping significant edges between masks was largest for the 2‐back and List Sorting masks, second largest for the 2‐back and Picture Sequence masks, and smallest for the 2‐back and Penn Word masks (see Table 6). Our identical analyses that used age‐adjusted rather than unadjusted Picture Sequence and List Sorting scores exhibited similar results (see Table S7).

**TABLE 6:** Functional anatomy similarity metric values

Training behaviors	Models trained using resting‐state functional connectivity		Models trained using *N*‐back task functional connectivity	
	Positive mask percentage of overlapping edges	Negative mask percentage of overlapping edges	Positive mask percentage of overlapping edges	Negative mask percentage of overlapping edges
2‐back/Penn Word	.04	.03	.06	.06
2‐back/Picture Sequence	.13	.10	.23	.20
2‐back/List Sorting	.17	.11	.71	.50

Note

For both positive and negative masks, the percentage of overlapping edges between the mask of significant edges used to predict the 2‐back score and the mask used to predict each other score. Larger percentages indicate greater similarity.

#### 3.3.2 Examining the brain connections used to predict each memory test score

**FIGURE 2:**
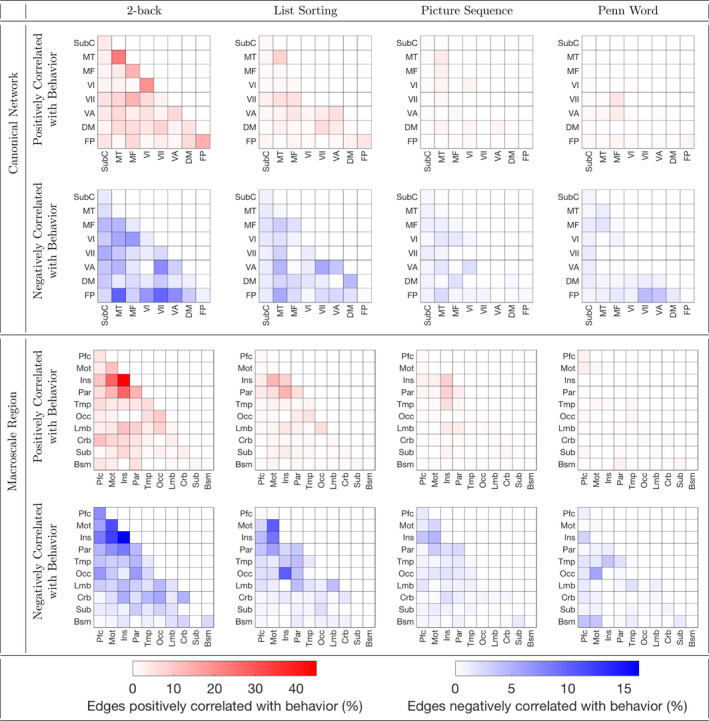
*N*‐back task functional connections predicting each memory test score. *N*‐back task functional connectivity positively (red) and negatively (blue) significantly related to each memory test score is grouped into canonical networks (top) and macroscale regions (bottom). Cells are shaded according to the percentage of all possible edges within a network/region or between a pair of networks/regions significantly related to performance on the task of interest (from left to right, 2‐back, List Sorting, Picture Sequence, or Penn Word). Canonical networks include the default mode (DM), subcortical cerebellum (SubC), frontoparietal (FP), motor (MT), medial frontal (MF), visual association (VA), VI, and VII. Macroscale regions include the prefrontal cortex (Pfc), motor cortex (Mot), insula (Ins), parietal (Par), temporal (Tmp), occipital (Occ), limbic (Lmb), cerebellum (Crb), subcortical (Sub), and brainstem (Bsm).

**FIGURE 3:**
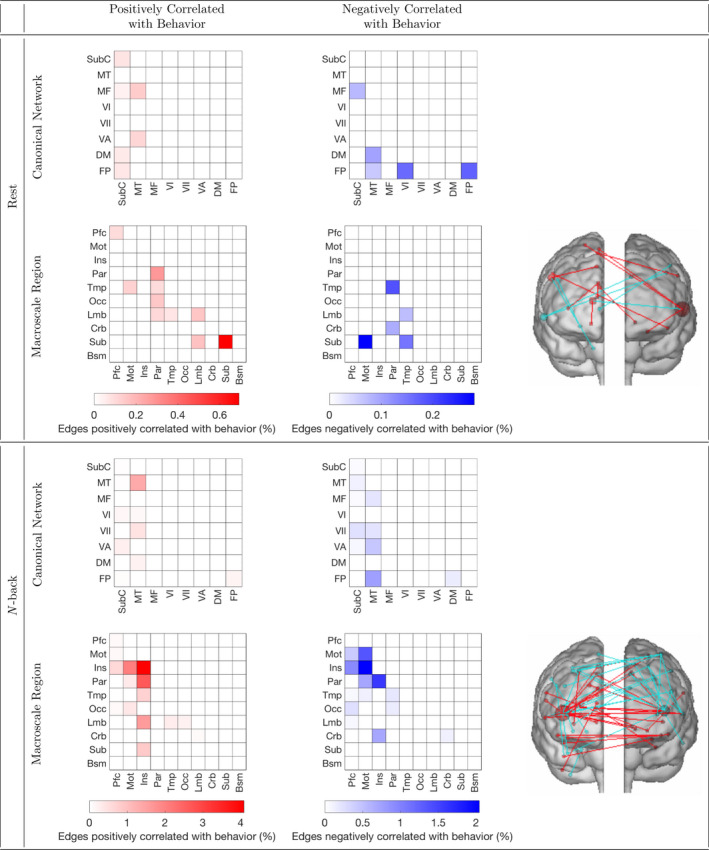
Functional connections predicting scores on the 2‐back, List Sorting, and Picture Sequence tasks. For each functional connection within a canonical network/macroscale region or between a pair of networks/regions, shading indicates the percentage of all possible rest (top) and *N*‐back task (bottom) edges that positively (red) and negatively (blue) significantly predict performance across the 2‐back, List Sorting, and Picture Sequence tasks. This figure examines the edges that significantly predicted all three of these scores, exploring which connections contribute to memory capabilities that are shared across these tasks. Glass brain plots (right) illustrate macroscale region connections that positively (red) and negatively (blue) predict behavior across all three tasks. Canonical networks include the default mode (DM), subcortical cerebellum (SubC), frontoparietal (FP), motor (MT), medial frontal (MF), visual association (VA), VI, and VII. Macroscale regions include the prefrontal cortex (Pfc), motor cortex (Mot), insula (Ins), parietal (Par), temporal (Tmp), occipital (Occ), limbic (Lmb), cerebellum (Crb), subcortical (Sub), and brainstem (Bsm).

**FIGURE 4:**
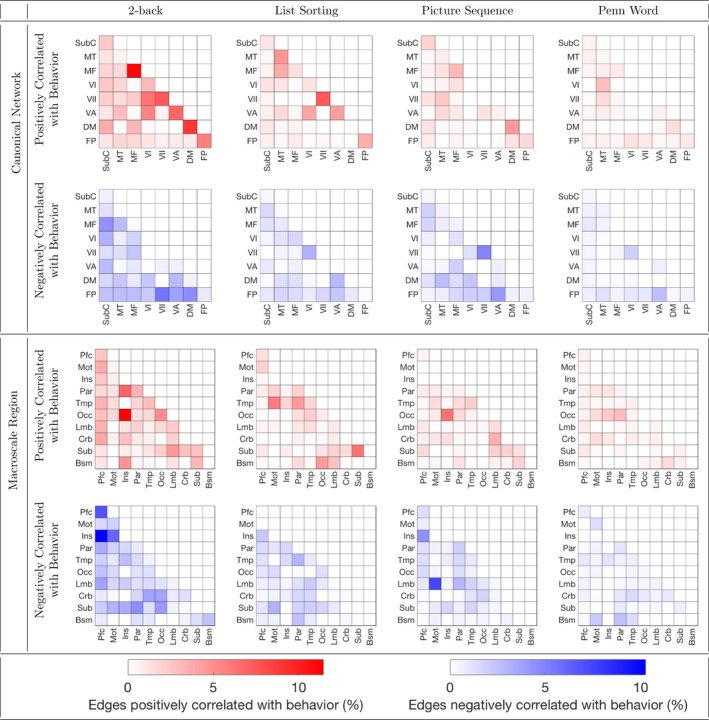
Rest functional connections predicting each memory test score. Rest functional connectivity positively (red) and negatively (blue) significantly related to each memory test score is grouped into canonical networks (top) and macroscale regions (bottom). Cells are shaded according to the percentage of all possible edges within a network/region or between a pair of networks/regions significantly related to performance on the task of interest (from left to right, 2‐back, List Sorting, Picture Sequence, or Penn Word). Canonical networks include the default mode (DM), subcortical cerebellum (SubC), frontoparietal (FP), motor (MT), medial frontal (MF), visual association (VA), VI, and VII. Macroscale regions include the prefrontal cortex (Pfc), motor cortex (Mot), insula (Ins), parietal (Par), temporal (Tmp), occipital (Occ), limbic (Lmb), cerebellum (Crb), subcortical (Sub), and brainstem (Bsm).

Figures 2 and 3 provide insight into the *N*‐back task functional connections predicting performance on all three of the 2‐back, List Sorting, and Picture Sequence tasks. Figure 2 separately visualizes the *N*‐back task functional connections significantly related to each memory test score. For both rest and *N*‐back task functional connectivity, Figure 3 visualizes the connections that significantly predicted all three of 2‐back, List Sorting, and Picture Sequence scores. *N*‐back task functional connectivity at least marginally significantly predicted all three of these scores. Thus, Figure 3 helps us understand which connections contribute to memory capabilities that are shared across these tasks. The *N*‐back task functional connectivity plots in Figure 3 and the plots in Figure 2 (2‐back: column 1, List Sorting: column 2, and Picture Sequence: column 3) illustrate an involvement of connections with the insula, including connections between insular and parietal regions, and between insular and motor regions. Figure 4 separately visualizes the rest functional connections significantly related to each memory test score. The rest functional connectivity plots in Figures 3 and 4 suggest that the predictive models based on rest functional connectivity do not all share these connections employed by the *N*‐back functional connectivity models. For plots showing age‐adjusted versions of the Picture Sequence and List Sorting scores produce similar trends, see Figures S4, S5, and S6.

## 4 DISCUSSION

In analyzing the extent to which *N*‐back task functional connectivity reflects different types of memory, we found that *N*‐back task functional connectivity predicts individual performance on another working memory measure, and, to a lesser extent, on an episodic memory measure. Furthermore, insular and parietal functional connectivity observed during *N*‐back task performance predicted both working and episodic memory. Together, these findings advance our understanding of relationships between working memory and episodic memory.

### 4.1 Analyzing the predictive power of rest and *N*‐back task functional connectivity for different memory test scores

Functional connectivity observed during *N*‐back task performance predicted individual differences in both working memory, measured with List Sorting memory test scores, and episodic memory, measured with Picture Sequence memory test scores, but predicted individual differences in working memory significantly better. The finding that the correlation between observed 2‐back and List Sorting scores was significantly greater than the correlation between observed 2‐back and Picture Sequence scores, as well as the sizable correlation between observed List Sorting and Picture Sequence scores, suggest that the results of these predictive analyses reflect behavioral‐level trends. The differences between the results of rest and *N*‐back task functional connectivity predictive analyses suggest that rest functional connectivity's predictive power for these memory test scores differs from that of *N*‐back task functional connectivity. Our *N*‐back task functional connectivity results demonstrate specificity, supporting the hypothesis that functional connectivity observed as a particular cognitive process is taxed may best predict that process (Finn et al., 2017). These results also, however, demonstrate generalizability, in that *N*‐back task functional connectivity reflects, to some degree, episodic memory abilities.

Our work, like Lugtmeijer et al. (2019) and Unsworth (2007), found a relationship between working and episodic memory capabilities for young adults. Like Lugtmeijer et al. (2019), we found a statistically significant relationship between *N*‐back test performance and episodic memory test performance for young adults. We also found that the correlations between the observed 2‐back, List Sorting, and Picture Sequence memory test scores of the HCP participants (see Table 1) were similar to the correlations between the same memory test scores for ages 9–10 Adolescent Brain Cognitive Development Study participants (Rosenberg, Martinez, et al., 2020). Thus, our findings may generalize to a younger age range.

Neither resting‐state nor *N*‐back task functional connectivity significantly predicted Penn Word scores. Thus, functional connectivity observed either during a working memory task or in the absence of a cognitive task did not predict individual differences in verbal episodic memory. The relatively small correlation between the observed Picture Sequence and Penn Word scores suggests that these two measures capture quite different types of episodic memory. Although, for Penn Word scores, we observe a null effect for functional connectivity observed both at rest and during a working memory task, it is an open question whether functional connectivity observed during a task that specifically engages episodic memory would predict individual differences in episodic memory ability.

### 4.2 Characterizing predictive network anatomy

Contributions of insular and parietal functional connectivity to predictions of 2‐back, List Sorting, and Picture Sequence scores align with previous results in the literature. For example, parietal activation is observed during both working memory tasks (Chai et al., 2018) and episodic retrieval (Cabeza et al., 2008). Vilberg and Rugg (2008) suggests that the episodic buffer, a part of Baddeley's working memory model that is critical to episodic long‐term memory (Baddeley, 2000), is located in the parietal cortex. In Baddeley's model, the episodic buffer is used to temporarily store multimodal information (Baddeley, 2000). Thus, these works suggest that maintenance of retrieved information could contribute to parietal activations for both working and episodic memory tasks. This hypothesis is consistent with Unsworth (2007), which suggests that those with lower working memory capacity experience deficits in episodic retrieval in part because they search through more items than those with strong working memory capacity do. Although the insula is not considered a core region consistently activated during working memory (Chai et al., 2018) or episodic memory (Allen & Fortin, 2013) tasks, previous work has found that the insula contributes to both working and episodic memory. Menon and Uddin (2010) found that the insula improves access to working memory resources during detection of an event. Xie et al. (2012) discovered that for individuals with amnestic mild cognitive impairment, disrupted intrinsic connectivity of the insula network is correlated with deficits in episodic memory. Rest functional connectivity 2‐back, List Sorting, and Picture Sequence plots did not all share these insular and parietal connections. Given that the *N*‐back functional connectivity models outperformed the rest functional connectivity models, these findings suggest that *N*‐back task functional connectivity captures more of the connections relevant to the memory capabilities shared by these tasks than rest functional connectivity does.

### 4.3 Limitations

One limitation of the present work is that age‐adjusted versions of scores were available only for the Picture Sequence and List Sorting scores, not for the 2‐back or Penn Word scores. Our analyses of age‐adjusted Picture Sequence and age‐adjusted List Sorting scores produced similar results to our analyses of the unadjusted versions of these scores. We do not believe that the unavailability of age‐adjusted versions of the other scores is a major issue as the age range of the examined HCP subjects is relatively narrow (mean age 28 ± 3.6 years).

Another potential limitation is that this work did not account for task‐related coactivation, which could have influenced connectivity estimates. Greene et al. (2020), which, like our paper, analyzed HCP data, found that for various tasks, task functional connectivity's ability to predict phenotype was not solely driven by task coactivation. This paper found that activation predicts phenotype only when the behavior completed during a task is related to the phenotype. Thus, it is possible that in our work, task coactivation accounts for some, but not all of the *N*‐back task functional connectivity's predictive power for related memory test scores. Additionally, our rest analysis found that rest functional connectivity marginally significantly predicted List Sorting test performance (*r* = .10, *p* = 4.8e−2). Previous work, such as Rosenberg et al. (2016) and Avery et al. (2020), also found that rest functional connectivity predicts behavioral score performance. These rest functional connectivity findings suggest that for *N*‐back task functional connectivity, task coactivation is not the sole predictor of memory test performance.

### 4.4 Future work

Further work could use similar techniques to examine how well *N*‐back task functional connectivity predicts other measures of working memory, episodic memory, and other types of memory. In evaluating how well *N*‐back task connectivity matrices can predict other working memory test scores, we could gain a deeper insight into the types of working memory that *N*‐back task functional connectivity captures well. Likewise, using *N*‐back task functional connectivity to predict test scores that primarily measure other types of memory could provide insight into the range of memory capabilities captured by *N*‐back task functional connectivity and the relationships between the functional connections predicting different types of memory.

## 5 CONCLUSIONS

Our work demonstrates the ability of *N*‐back task functional connectivity to predict individual performance on another measure of working memory, and, to a lesser extent, on a measure of episodic memory. Thus, *N*‐back task functional connectivity may capture cognitive processes that are essential to both working and episodic memory. By furthering our knowledge of *N*‐back task functional connectivity, these findings provide insights into relationships between working memory and episodic memory, which are critical to better understanding many neurological conditions and crucial cognitive abilities.

## CONFLICT OF INTEREST

The authors have no conflicts of interest to declare.

## AUTHOR CONTRIBUTION

G.F.S., E.W.A., K.Y., and M.M.C. conceptualized the study. A.S.G., S.G., D.S., and R.T.C. processed the data. G.F.S. performed analyses with support from E.W.A., K.Y., and M.D.R.. All authors provided guidance on result interpretation and follow‐up analysis design. G.F.S. wrote the manuscript with contributions from E.W.A., K.Y., M.D.R., and M.M.C.. All authors reviewed and commented on the final manuscript.

## Supporting information

Figure S1

Figure S2

Figure S3

Figure S4

Figure S5

Figure S6

Table S1‐S7

## Data Availability

Data were provided by the Human Connectome Project, WU‐Minn Consortium (Principal Investigators: David Van Essen and Kamil Uğurbil; 1U54MH091657) funded by the 16 NIH Institutes and Centers that support the NIH Blueprint for Neuroscience Research; and by the McDonnell Center for Systems Neuroscience at Washington University. The Human Connectome Project data are available for download at www.humanconnectome.org. All code is available from the corresponding author upon request.
