# Supplementary material for: Using functional connectivity models to characterize relationships between working and episodic memory
Source: Brain Behav. 2021 Jun 17;11(8):e02105. doi: 10.1002/brb3.2105 (PMC8413720; doi:10.1002/brb3.2105)
Supplement: Supplementary file 1 — Figure S1 [file BRB3-11-e02105-s002.pdf]

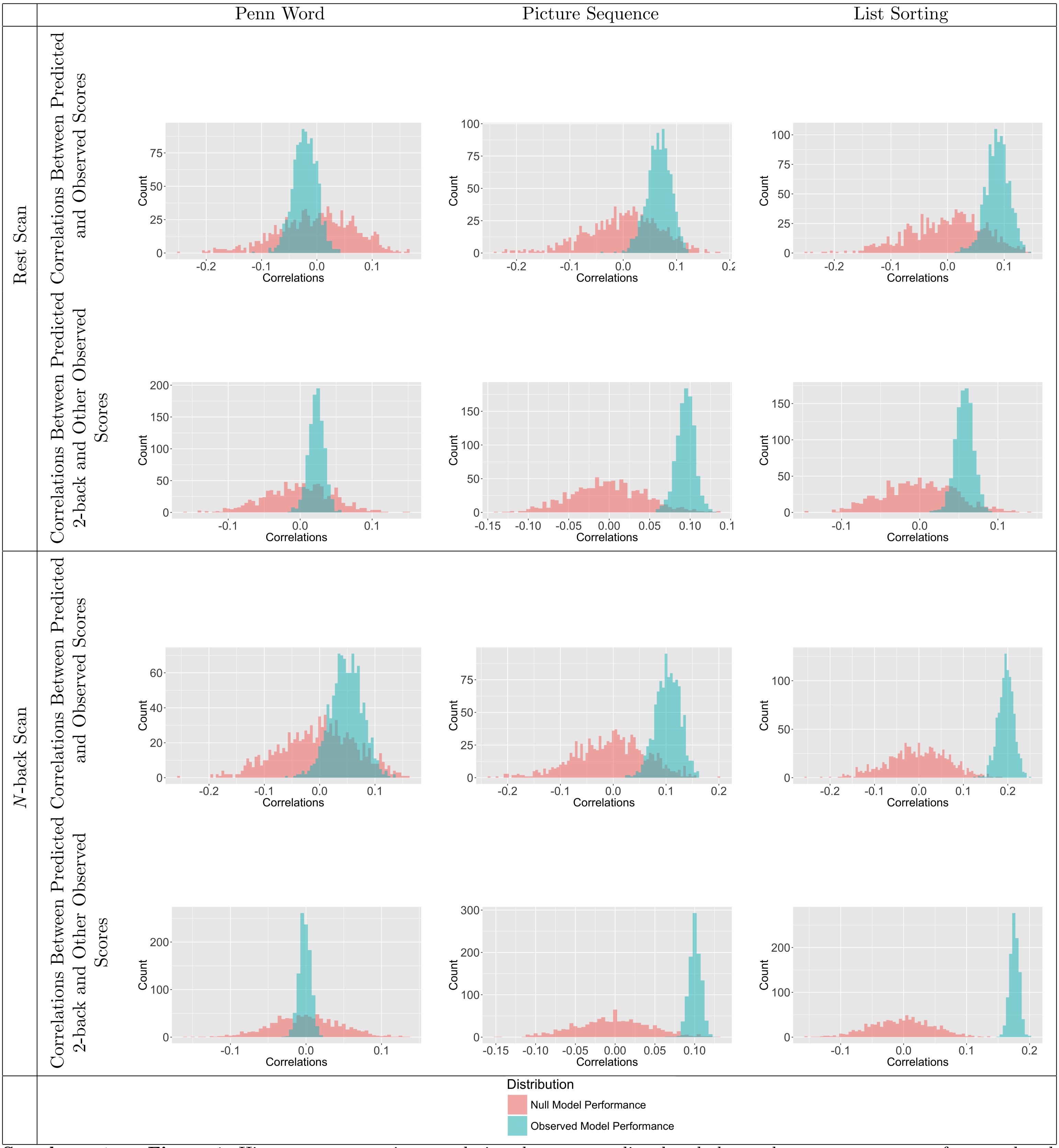

Supplementary Figure 1. Histograms comparing correlations between predicted and observed memory test scores for actual and null connectome-based predictive models (age-adjusted). For both rest and N-back task functional connectivity and for iterations of both actual and null connectome-based predictive models (CPMs), each of the 1000 correlations between predicted and observed Penn Word, age-adjusted Picture Sequence, and age-adjusted List Sorting scores and each of the 1000 correlations between predicted 2-back and observed Penn Word, age-adjusted Picture Sequence, and age-adjusted List Sorting scores.
