# Supplementary material for: Using functional connectivity models to characterize relationships between working and episodic memory
Source: Brain Behav. 2021 Jun 17;11(8):e02105. doi: 10.1002/brb3.2105 (PMC8413720; doi:10.1002/brb3.2105)
Supplement: Supplementary file 3 — Figure S3 [file BRB3-11-e02105-s007.pdf]

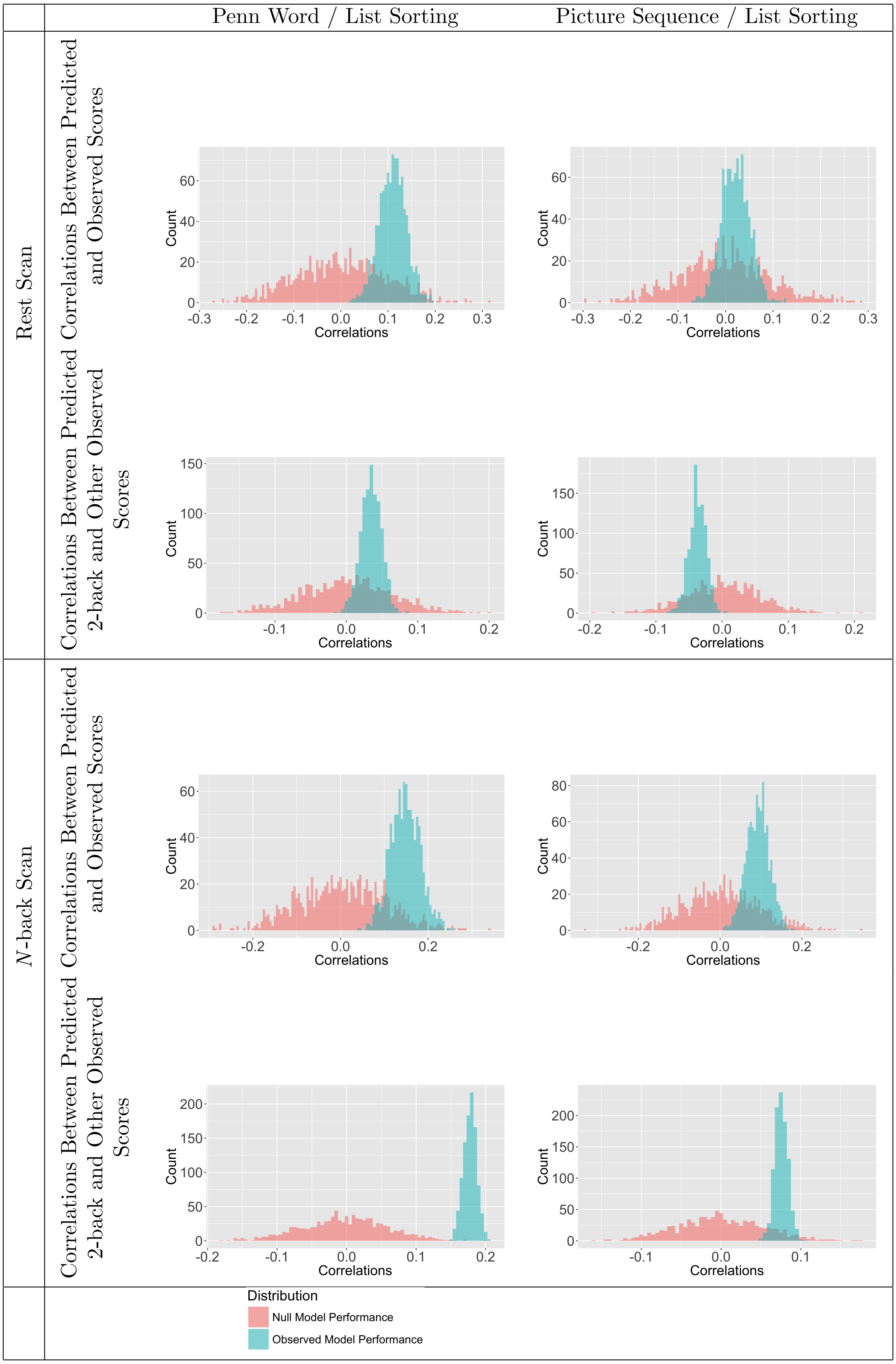

Supplementary Figure 3. Histograms comparing differences between correlations between predicted and observed memory test scores for actual and null connectome-based predictive models (age-adjusted). For both rest and N-back task functional connectivity and for iterations of both actual and null connectome-based predictive models (CPMs): 1) a distribution of correlations between predicted and observed age-adjusted List Sorting memory test scores minus correlations between predicted and observed Penn Word memory test scores, 2) a distribution of correlations between predicted and observed age-adjusted List Sorting memory test scores minus correlations between predicted and observed age-adjusted Picture Sequence memory test scores, 3) a distribution of correlations between predicted 2-back and observed age-adjusted List Sorting memory test scores minus correlations between predicted 2-back and observed Penn Word memory test scores, and 4) a distribution of correlations between predicted 2-back and observed age-adjusted List Sorting memory test scores minus correlations between predicted 2-back and observed age-adjusted Picture Sequence memory test scores.
