# Supplementary material for: Using functional connectivity models to characterize relationships between working and episodic memory
Source: Brain Behav. 2021 Jun 17;11(8):e02105. doi: 10.1002/brb3.2105 (PMC8413720; doi:10.1002/brb3.2105)
Supplement: Supplementary file 5 — Figure S5 [file BRB3-11-e02105-s005.pdf]

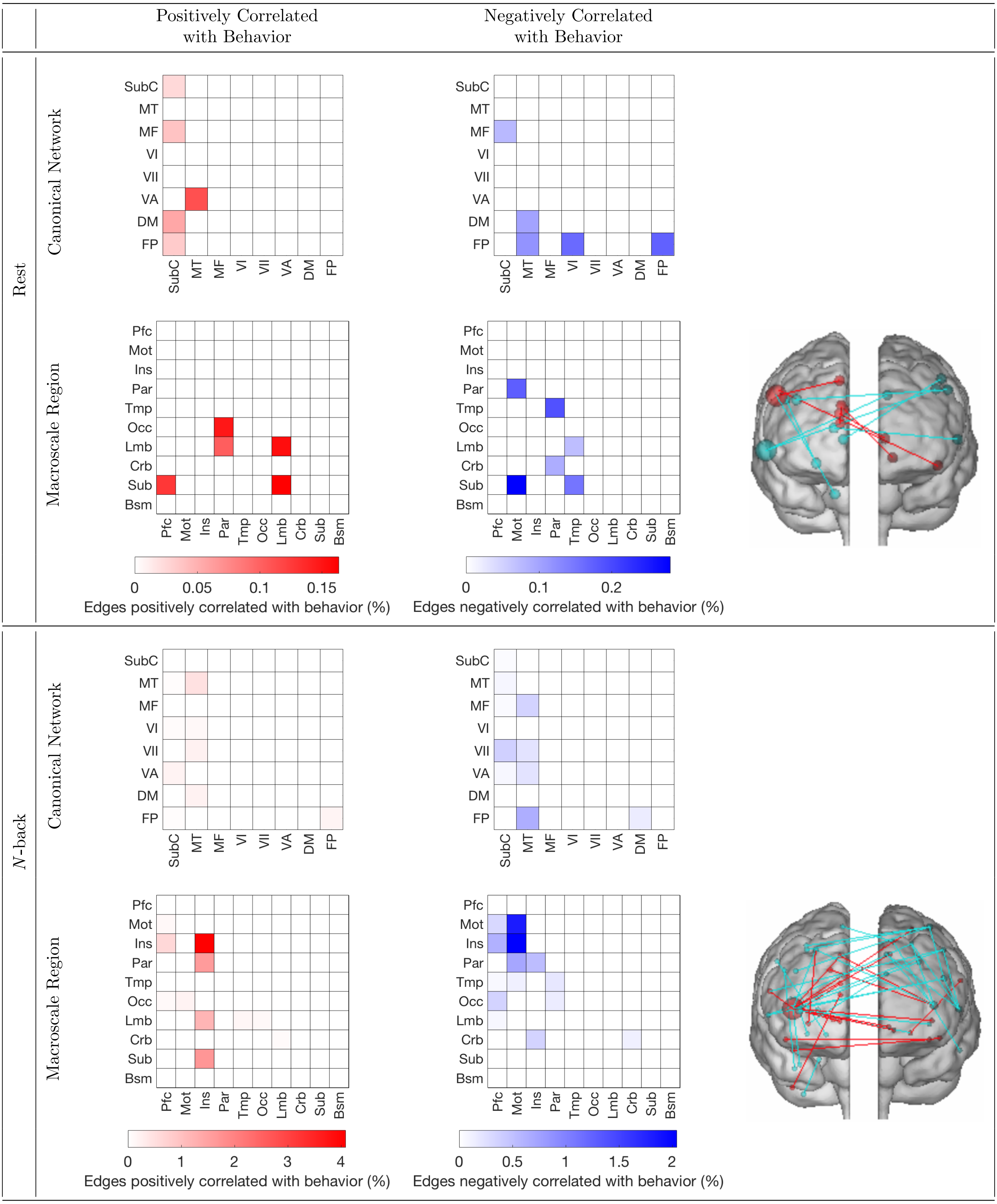

Supplementary Figure 5. Functional connections predicting scores on the 2-back, List Sorting, and Picture Sequence tasks (age-adjusted). For each functional connection within a canonical network / macroscale region or between a pair of networks / regions, shading indicates the percentage of all possible rest (top) and N-back task (bottom) edges that positively (red) and negatively (blue) significantly predict performance across the 2-back, age-adjusted List Sorting, and age-adjusted Picture Sequence tasks. This figure examines the edges that significantly predicted all three of these scores, exploring which connections contribute to memory capabilities that are shared across these tasks. Glass brain plots (right) illustrate macroscale region connections that positively (red) and negatively (blue) predict behavior across all three tasks. Canonical networks include the default mode (DM), subcortical cerebellum (SubC), frontoparietal (FP), motor (MT), medial frontal (MF), visual association (VA), VI, and VII. Macroscale regions include the prefrontal cortex (Pfc), motor cortex (Mot), insula (Ins), parietal (Par), temporal (Tmp), occipital (Occ), limbic (Lmb), cerebellum (Crb), subcortical (Sub), and brainstem (Bsm).
