# Supplementary material for: Using functional connectivity models to characterize relationships between working and episodic memory
Source: Brain Behav. 2021 Jun 17;11(8):e02105. doi: 10.1002/brb3.2105 (PMC8413720; doi:10.1002/brb3.2105)
Supplement: Supplementary file 6 — Figure S6 [file BRB3-11-e02105-s006.pdf]

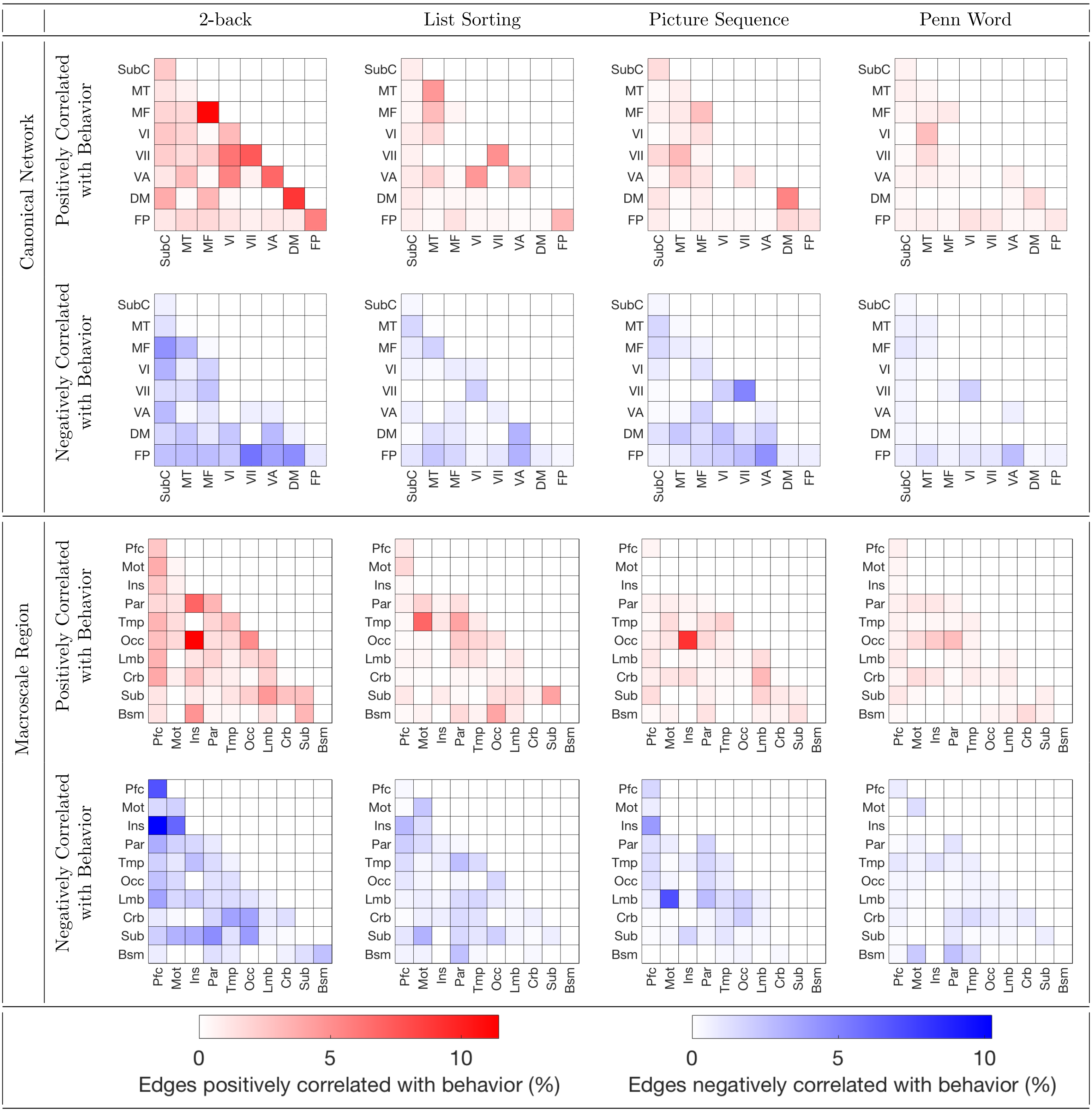

Supplementary Figure 6. Rest functional connections predicting each memory test score (age-adjusted). Rest functional connectivity positively (red) and negatively (blue) significantly related to each memory test score is grouped into canonical networks (top) and macroscale regions (bottom). Cells are shaded according to the percentage of all possible edges within a network / region or between a pair of networks / regions significantly related to performance on the task of interest (from left to right, 2-back, age-adjusted List Sorting, age-adjusted Picture Sequence, or Penn Word). Canonical networks include the default mode (DM), subcortical cerebellum (SubC), frontoparietal (FP), motor (MT), medial frontal (MF), visual association (VA), VI, and VII. Macroscale regions include the prefrontal cortex (Pfc), motor cortex (Mot), insula (Ins), parietal (Par), temporal (Tmp), occipital (Occ), limbic (Lmb), cerebellum (Crb), subcortical (Sub), and brainstem (Bsm).
