# Supplementary material for: Using functional connectivity models to characterize relationships between working and episodic memory
Source: Brain Behav. 2021 Jun 17;11(8):e02105. doi: 10.1002/brb3.2105 (PMC8413720; doi:10.1002/brb3.2105)
Supplement: Supplementary file 7 — Table S1‐S7 [file BRB3-11-e02105-s004.pdf]

Supplementary Tables

Supplementary Table 1. Correlations between observed memory test scores (age-adjusted)

	Penn Word	*Picture Sequence†*	*List Sorting†*
2-back	.20 (*p*=5.2e-6)	.25 (*p*=1.0e-8)	.36 (*p*<2.2e-16)
Penn Word	-	.20 (*p*=6.3e-6)	.08 (*p*=7.9e-2)
Picture Sequence†	-	-	.30 (*p*=7.0e-12)

The correlations between the observed 2-back, Penn Word, Picture Sequence, and List Sorting memory test scores of the 502 examined Human Connectome Project (HCP) participants.
†These scores are age-adjusted.

Supplementary Table 2. Steiger's *z*-test results comparing correlations between observed memory test scores (age-adjusted)

**Observed behaviors**	***z*-value**	***p*-value**
2-back / Picture Sequence† & 2-back / List Sorting†	2.2	2.9e-2
2-back / Penn Word & 2-back / List Sorting†	2.8	5.5e-3

The Steiger's *z*-test *z*-values and *p*-values for comparisons of the correlations between observed memory test scores.
†These scores are age-adjusted.

Supplementary Table 3. Correlations between predicted and observed memory test scores (age-adjusted)

	**Models trained and tested using resting-state functional connectivity**			**Models trained and tested using *N*-back task functional connectivity**		
**Training & test behavior**	**Mean *r*-value**	**Standard deviation *r*-value**	***p*-value**	**Mean *r*-value**	**Standard deviation *r*-value**	***p*-value**

2-back	.20‡		1/1001‡	.36‡		1/1001‡
Penn Word	-.02	.02	.62	.05	.03	.24
Picture Sequence†	.07	.02	.16	.10	.02	4.6e-2
List Sorting†	.09	.02	7.0e-2	.20	.02	1.0e-3

The correlations between the predicted and observed Penn Word, Picture Sequence, and List Sorting memory test scores of the 502 examined Human Connectome Project (HCP) participants.

†These scores are age-adjusted.

‡These numbers were taken from Avery et al. (2020), which for the same set of subjects as our work, used the same rest and *N*-back task functional connectivity and 10-fold cross validation to predict 2-back memory test scores.

Supplementary Table 4. Correlations between predicted 2-back and other observed memory test scores (age-adjusted)

	**Models trained and tested using resting-state functional connectivity**			**Models trained and tested using *N*-back task functional connectivity**		
**Observed behavior**	**Mean *r*-value**	**Standard deviation *r*-value**	***p*-value**	**Mean *r*-value**	**Standard deviation *r*-value**	***p*-value**
Penn Word	.02	.01	.31	-1.6e-3	7.4e-3	.51
Picture Sequence†	.09	.01	2.2e-2	.10	.01	1.5e-2
List Sorting†	.06	.01	9.6e-2	.18	.01	1.0e-3

The correlations between the predicted 2-back and the observed Penn Word, Picture Sequence, and List Sorting memory test scores of the 502 examined Human Connectome Project (HCP) participants.

†These scores are age-adjusted.

Supplementary Table 5. Comparing correlations between predicted and observed memory test scores (age-adjusted)

	Models trained and tested using resting-state functional connectivity			Models trained and tested using *N*-back task functional connectivity		
**Training & test behaviors**	***t*-value**	***p*-value**	**97.5% confidence interval**	***t*-value**	***p*-value**	**97.5% confidence interval**
Penn Word / List Sorting†	34.7	<2.2e-16	(.10, .12)	44.2	<2.2e-16	(.14, .15)
Picture Sequence† / List Sorting†	7.6	8.3e-14	(.02, .03)	29.4	<2.2e-16	(.08, .10)

The *t*-test results for comparing difference distributions for the correlations between predicted and observed memory test scores to the corresponding null difference distributions. Each actual and each null difference distribution equals a distribution of correlations between predicted and observed List Sorting scores minus correlations between predicted and observed Picture Sequence or Penn Word scores. Thus, a significant positive *t*-value indicates that List Sorting scores were predicted significantly better than Picture Sequence or Penn Word scores. A significant negative *t*-value indicates that Picture Sequence or Penn Word scores were predicted significantly better than List Sorting scores.
†These scores are age-adjusted.

Supplementary Table 6. Comparing correlations between predicted 2-back and other observed memory test scores (age-adjusted)

	Models trained and tested using resting-state functional connectivity			Models trained and tested using *N*-back task functional connectivity		
**Observed behaviors**	***t*-value**	***p*-value**	**97.5% confidence interval**	***t*-value**	***p*-value**	**97.5% confidence interval**
Penn Word / List Sorting†	16.6	<2.2e-16	(.03, .04)	92.9	<2.2e-16	(.18, .18)
Picture Sequence† /	-21.5	<2.2e-16	(-.04, -.03)	43.9	<2.2e-16	(.07, .08)

List Sorting†						

The *t*-test results for comparing difference distributions for the correlations between predicted 2-back and other observed memory test scores to the corresponding null difference distributions. Each actual and each null difference distribution equals a distribution of correlations between predicted 2-back and observed List Sorting scores minus correlations between predicted 2-back and observed Picture Sequence or Penn Word scores. Thus, a significant positive *t*-value indicates that CPM-predicted 2-back scores are significantly more similar to observed List Sorting scores than to observed Picture Sequence or Penn Word scores. A significant negative *t*-value indicates that CPM-predicted 2-back scores are significantly more similar to observed Picture Sequence or Penn Word scores than to observed List Sorting scores.
†These scores are age-adjusted.

Supplementary Table 7. Functional anatomy similarity metric values (age-adjusted)

	**Models trained using resting-state functional connectivity**		**Models trained using *N*-back task functional connectivity**	
**Training behaviors**	**Positive mask percentage of overlapping edges**	**Negative mask percentage of overlapping edges**	**Positive mask percentage of overlapping edges**	**Negative mask percentage of overlapping edges**
2-back / Penn Word	.04	.03	.06	.06
2-back / Picture Sequence†	.12	.10	.17	.17
2-back / List Sorting†	.13	.11	.52	.41

For both positive and negative masks, the percentage of overlapping edges between the mask of significant edges used to predict the 2-back score and the mask used to predict each other score. Larger percentages indicate greater similarity.
†These scores are age-adjusted.
